# Losartan Improved Antioxidant Defense, Renal Function and Structure of Postischemic Hypertensive Kidney

**DOI:** 10.1371/journal.pone.0096353

**Published:** 2014-05-05

**Authors:** Milan Ivanov, Nevena Mihailović-Stanojević, Jelica Grujić Milanović, Đurđica Jovović, Jasmina Marković-Lipkovski, Sanja Ćirović, Zoran Miloradović

**Affiliations:** 1 Department of Cardiovascular Physiology, Institute for Medical Research, University of Belgrade, Belgrade, Serbia; 2 Institute of Pathology, Medical Faculty, University of Belgrade, Belgrade, Serbia; National Cancer Institute, United States of America

## Abstract

Ischemic acute renal failure (ARF) is a highly complex disorder involving renal vasoconstriction, filtration failure, tubular obstruction, tubular backleak and generation of reactive oxygen species. Due to this complexity, the aim of our study was to explore effects of Angiotensin II type 1 receptor (AT1R) blockade on kidney structure and function, as well as oxidative stress in spontaneously hypertensive rats (SHR) after renal ischemia reperfusion injury. Experiments were performed on anaesthetized adult male SHR in the model of ARF with 40 minutes clamping the left renal artery. The right kidney was removed and 40 minutes renal ischemia was performed. Experimental groups received AT1R antagonist (Losartan) or vehicle (saline) in the femoral vein 5 minutes before, during and 175 minutes after the period of ischemia. Biochemical parameters were measured and kidney specimens were collected 24h after reperfusion. ARF significantly decreased creatinine and urea clearance, increased LDL and lipid peroxidation in plasma. Treatment with losartan induced a significant increase of creatinine and urea clearance, as well as HDL. Lipid peroxidation in plasma was decreased and catalase enzyme activity in erythrocytes was increased after losartan treatment. Losartan reduced cortico-medullary necrosis and tubular dilatation in the kidney. High expression of pro-apoptotic Bax protein in the injured kidney was downregulated after losartan treatment. Our results reveal that angiotensin II (via AT1R) mediates the most postischemic injuries in hypertensive kidney through oxidative stress enhancement. Therefore, blockade of AT1R may have beneficial effects in hypertensive patients who have developed ARF.

## Introduction

Acute renal failure (ARF) has a multifactorial causality, but the mechanism of pathogenesis and the development of this disease are still incompletely defined. Ischemia/reperfusion injury (IRI) frequently occurs in several clinical situations including renal transplantation, suprarenal procedures of aorta and certain hypotensive states [1].

It is known from experimental studies that both ischemia and reinstitution of blood flow in the kidney injury generate reactive oxygen species (ROS), during and after reperfusion. Under normal conditions, antioxidant defense system neutralizes cellular effects of oxygen-free radicals, but due to reperfusion in ischemic tissue, the protective capacity of these scavengers is overwhelmed by rapid generation of ROS. Renal dysfunction caused by overproduction of ROS in IRI is closely associated with cell membrane peroxidation, mitochondrial dysfunction, inhibition of protein synthesis, DNA damage and inhibition of the antioxidant system [2], [3]. Therefore, the main target of IRI therapies should be found in the pharmacological approach for decreasing the renal oxidative stress.

Angiotensin II (Ang II), as one of the main vasoactive signaling molecule, is involved in the generation of ROS. Overproduction of Ang II during the ischemic ARF [4], [5] participates in increased expression and activity of one of the major ROS generators, NADPH oxidase [6]. Also, it is well known that the increased production of Ang II occurs in hypertensive conditions [7]. Beyond intrarenal vasoconstriction, high level of Ang II has harmful effect on necrotic and apoptotic changes in kidney tissue during reperfusion period, as well [3], [8]. Furthermore, Ang II down-regulates the SR-BI HDL receptor in proximal tubular cells [9], therefore abolishes the protective HDL effects on postischemic kidney [10].

Ratio between expression of pro-apoptotic (i.e., Bax) and anti-apoptotic (i.e., Bcl-2) genes is crucial for cell surviving [11]. During kidney IRI, expression of these proteins in renal tissue is disturbed [3]. Further, it has been reported that Losartan has relevance on Bax and Bcl-2 expression in hypertension [11].

Taking all together, the goal of our research was to explain a relation between the increased oxidative stress and blockade of Ang II type 1 receptor (AT1R) receptor during the postischemic ARF in hypertension.

## Materials and Methods

In our study we used 24 weeks old male spontaneously hypertensive rats (SHR), weighing about 300 g, which were bred at the Institute for Medical Research, University of Belgrade, Serbia, and fed with a standard chow for laboratory rats (Veterinarski zavod, Subotica, Serbia).

### Ethics Statement

The experimental protocol was approved by the Ethic Committee of the Institute for Medical Research, University of Belgrade, Serbia (No. 0148.1/10), according to the National Law on Animal Welfare ("Službeni Glasnik" no. 41/09) that is consistent with guidelines for animal research and principles of the European Convention for the Protection of Vertebrate Animals Used for Experimental and Other Purposes (Official Daily N. L 358/1-358/6, 18, December 1986) and Directive on the protection of animals used for scientific purposes (Directive 2010/63/EU of the European Parliament and of the Council, 22.9.2010.).

### Experimental protocol

Animals were divided into the following groups: sham-operated rats (SHAM, n = 7), rats with ARF (ARF, n = 7) and animals received AT1R antagonist Losartan (DUP 153, Du Pont, Wilmington, DE, USA) after ARF induction (ARF+LOS, n = 9).

For surgical procedure, all rats were anaesthetized with 35 mg/kg b.m. sodium pentobarbital. ARF was induced by right kidney nephrectomy and atraumatic occlusion of left renal artery for 40 minutes. SHAM and ARF rats received vehicle (saline, 4 ml) infusion, while ARF+LOS group received Losartan (10 mg/kg b.m dissolved in 4 ml saline) infusion, in the femoral vein 5 minutes before, during and 175 minutes after clamping. After infusion, abdominal incision was closed by several sutures and SHR were placed into metabolic cages for 24 h urine collection, having free access to water and chow.

### Blood pressure measurements

Mean arterial pressure (MAP) was measured in anaesthetized rats. Left femoral artery was canulated by PE–50 catheter (Clay-Adams Parsippany, NY, USA) and connected to the physiological data acquisition system (9800TCR Cardiomax III-TCR, Columbus, OH, USA). Measurements were performed before and 24 hours after reperfusion.

### Biochemical measurements 24 h after reperfusion

After 24 h urine collection all animals were anaesthetized (35 mg/kg b.m. sodium pentobarbital, i.p.) and blood samples were taken for determination of creatinine (P_Cr_), urea (P_U_), cholesterol, HDL and triglyceride levels in plasma. Lithium-heparin (Li-heparin, Sigma, USA) was used as an anticoagulant. 24 h urine samples were used for determination of urine creatinine (U_Cr_) and urea (U_U_) concentrations. All biochemical parameters were measured using an automatic COBAS INTEGRA 400 plus (Hoffmann-La Roche, Germany) analyzer. LDL was calculated from Friedewald equation. Creatinine (C_Cr_) and urea (C_U_) clearances were calculated according to standard formula and normalized to body weight. After blood samples collection, animals were sacrificed by pentobarbital overdose injection.

### Oxidative stress parameters

In order to determine the degree of lipid peroxidation (LP), the concentration of thiobarbituric acid reactive substances (TBARS) in plasma was measured [12]. Erythrocytes catalase (CAT), glutathione reductase (GR), superoxide dismutase (SOD) and glutathione peroxidase (GSH-Px) activities were measured by spectrophotometry, according to previously described methods [13]–[16].

### Morphological and immunohistological examination

At the moment of sacrificing, the left kidney was taken for the morphological analysis. Renal tissue was prepared as described previously [8], and stained by hematoxiline eosine (H&E) and periodic acid-Schiff (PAS). Intensity and spread of tubular necrosis, number of intra-luminal cast formations, swelling and vacuolization of cells, loss of luminal membrane or brush borders, tubular dilatation, interstitial oedema and separation of cells from tubular basal membrane were semi-quantitatively evaluated as described previously [8]. The level of each manifestation was graded with 1 for low, 2 for moderate, 3 for high, and 0 for the lack of manifestation. The sum of these changes represented the histopathological score.

For investigation of Bax and Bcl-2 expression paraffin, sections were treated by microwave for 20 min at 400 W in citrate buffer (pH 6.0) after deparaffinization and dehydration. After antigen retrieval, samples were incubated with Bax (dilution 1∶250, Millipore, Billerica, MA) and Bcl-2 (dilution 1∶200, Millipore, Billerica, MA) antibodies for 1 hour at room temperature. The EnVisionTM staining method (DAKO) was performed, followed by counterstaining with hemalaun (Merck). Negative controls were performed by omitting the first antibody. The slides were evaluated using the light microscope BX53 (Olympus).

### Statistical analysis

The results are shown as the mean with the standard error of the mean. We used the single-sided Student's t-test for two-samples of equal variance, and value p<0.05 was considered notable (Microsoft Excel 2010).

## Results

### Blood pressure measurements

**Figure 1:**
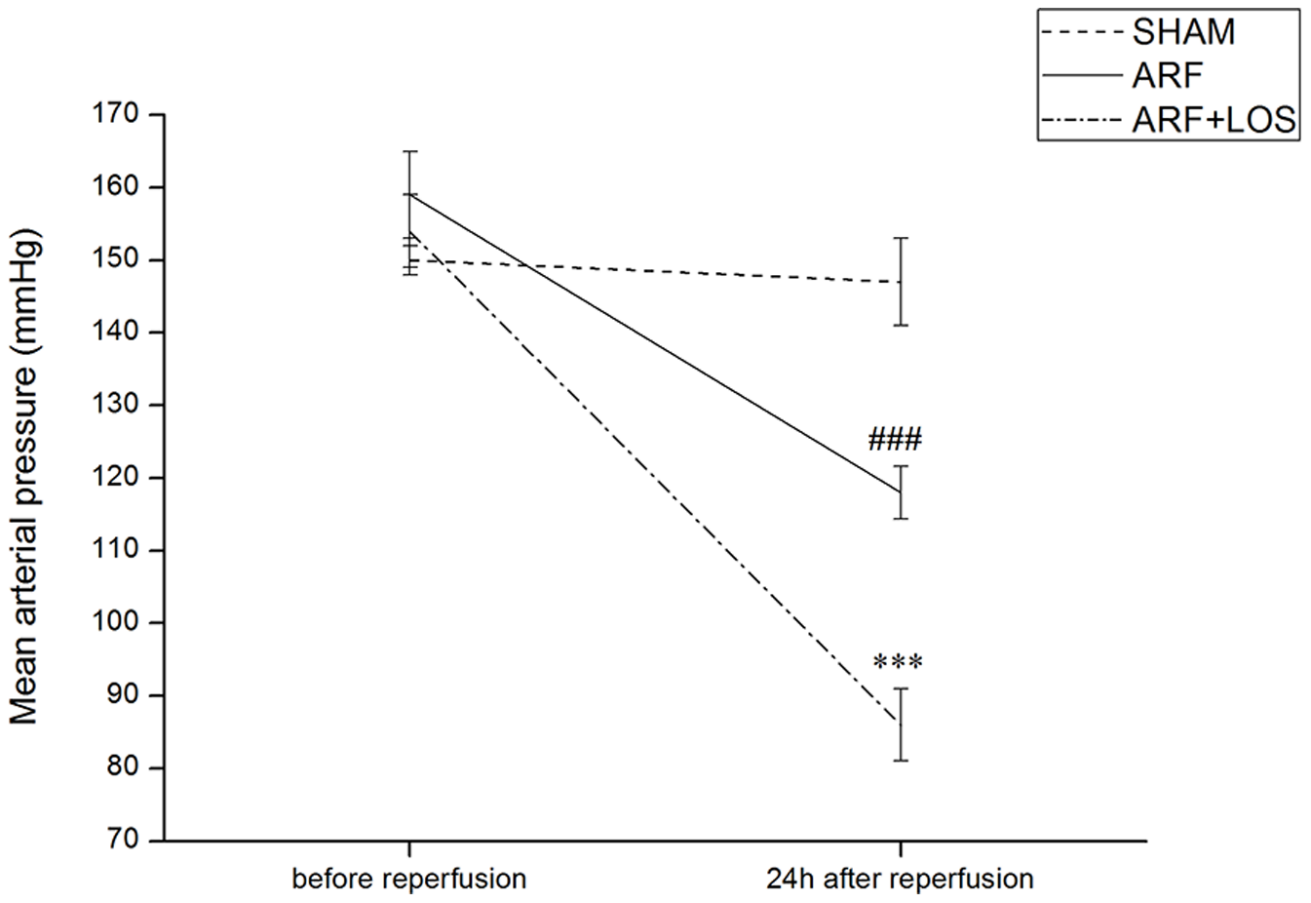
Mean arterial pressure in experimental groups before and 24 h after reperfusion. SHAM, n = 7; ARF, n = 7; ARF+LOS, n = 9 (n-number of animals). Data are presented as mean ± SEM (###p<0.001 compared with SHAMl; ***p<0.001 compared with ARF).

There was no significant MAP difference between groups before ARF induction. MAP was significantly decreased in ARF compared to SHAM as well as in ARF+LOS vs. ARF group 24 hours after reperfusion (Figure 1).

### Biochemical parameters

Biochemical parameters are presented in **Table 1**. C_Cr_ and C_U_ were significantly lower in ARF group vs. SHAM. Treatment with Losartan markedly increased C_Cr_ and C_U_ in comparison to ARF group. There were non-significant differences between triglycerides and total cholesterol level in experimental groups. Plasma LDL was significantly increased in ARF group compared to SHAM, and Losartan reduced it, not significantly (p = 0.054), but almost near a value as in SHAM. HDL cholesterol was significantly higher in ARF+LOS group, compared to level in ARF group.

**Table 1:** Biochemical parameters in experimental groups 24/reperfusion injury.

	SHAM (n = 7)	ARF (n = 7)	ARF+LOS (n = 9)
**Creatinine clearances (ml/min/kg)**	6.50±0.99	0.29±0.13^###^	1.70±0.20***
**Urea clearances (ml/min/kg)**	2.38±0.28	0.10±0.05^###^	0.64±0.09***
**Cholesterol (mmol/l)**	1.32±0.09	1.53±0.09	1.53±0.12
**Triglycerides (mmol/l)**	0.89±0.10	0.86±0.08	0.97±0.08
**HDL (mmol/l)**	0.61±0.03	0.57±0.05	0.75±0.02**
**LDL (mmol/l)**	0.3±0.08	0.57±0.08#	0.34±0.10

# *p*<0.05,^ ###^ *p*<0.001 compared with SHAM level; **p<0.01, ***p<0.001 compared with ARF. Data are presented as mean ± SEM. n-number of animals.

### Oxidative stress parameters

SOD, GSH-Px and GR activity, were not different between groups (**Table 2**). ARF group showed significantly higher TBARS level compared to SHAM operated animals, with marked, but not significant, decrease of CAT activity. Losartan infusion resulted in both a significant increase of CAT activity and the decline of TBARS compared to ARF alone (**Table 2**).

**Table 2:** Oxidative stress parameters in experimental groups 24 h after reperfusion.

	SHAM (n = 7)	ARF (n = 7)	ARF+LOS (n = 9)
**pTBARS (nmol/ml)**	7.28±0,76	10.54±0.52##	7.21±0.36**
**eCAT (mmol H_2_O_2_/min)**	22.15±6.05	14.32±2.91	22.57±3.25*
eGR (µmol NADPH/min/g Hb) *10^3^	5.6±1.8	4.9±1.2	7.4±2.2
**eGSH-Px (µmol NADPH/min/g Hb)**	185.1±59.4	152.44±27.1	188.6±31.1
eSOD (U/g Hb)*10^3^	1.6±0.4	1.5±0.5	2.1±0.3

## *p*<0.01 compared with SHAM level; *p<0.05, **p<0.01 compared with ARF. Data are presented as mean ± SEM; n-number of animals; p-plasma; e- erythrocyte.

### Histological studies

**Figure 2:**
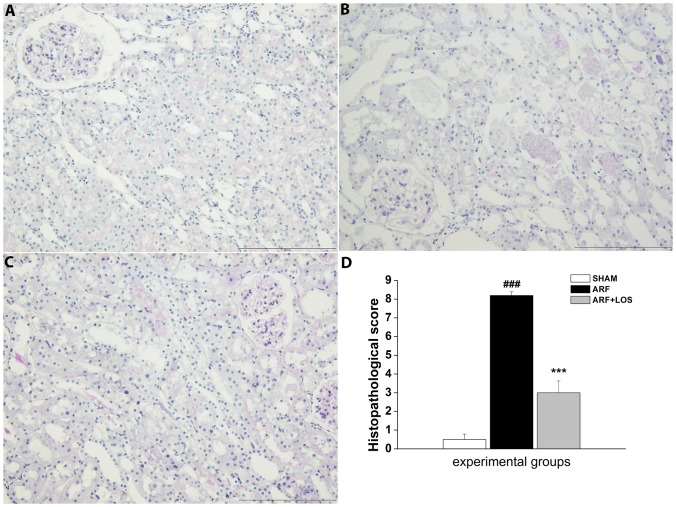
Histology of the kidney 24 h after reperfusion. (**A**) normal shape of the glomerulus and tubulointerstitium in the sham-operated animals (**B**) proximal tubular dilatation and necrosis, PAS-positive casts in the ARF control group (**C)** moderately intensive tubular necrosis in Losartan treated rats, reduced tubular dilatation and less number of PAS-positive casts (**D**) Histopathological score in experimental groups 24 h after reperfusion. SHAM, n = 7; ARF, n = 7; ARF+LOS, n = 9 (n-number of animals). Data are presented as mean ± SEM (^###^p<0.001 compared with SHAMl; ***p<0.001 compared with ARF).

There were significant differences in pathomorphological parameters between experimental groups. **Figure 2A** shows the normal appearance of glomeruli, interstitium, tubules and blood vessels in SHAM operated animals. Only in a few kidney specimens there was a less number of PAS positive casts in the lumen of the tubules. The kidneys of animals with ARF showed dilatation of certain segments of the proximal and distal tubules, with or without loss of brush-border. The most notable changes were present in the cortico-medullary zone, where the broad areas of tubular necrosis and a large number of PAS positive casts in the collecting ducts were observed. The intensity of interstitial edema in this group varies from sample to sample (**Figure 2B**). In Losartan treated animals, less damage is noticed in comparison to renal tissue of ARF animals. Tubular dilatation is smaller or even absent in some kidney specimens. In the cortico-medullary zone, tubular necrosis is reduced. Interstitial edema is rarely observed. In addition, the number of tubular casts in the renal medulla is lower, compared to ARF animals (**Figure 2C**). Histopathological score was shown in **Figure 2D**.

**Figure 3:**
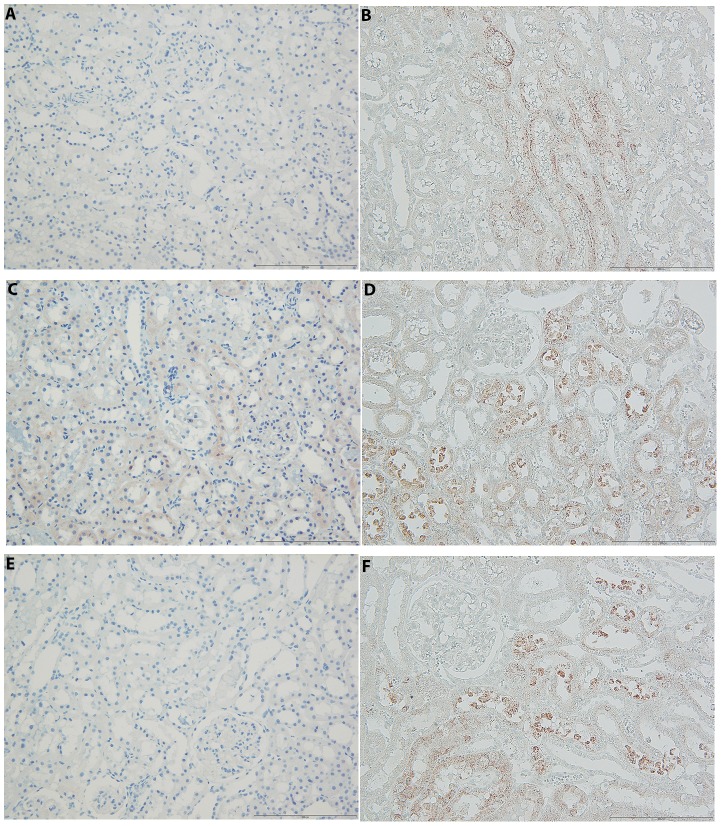
Bcl-2 and Bax expression in kidney tissue 24 h after reperfusion. (**A**) almost absence of Bcl-2 expression on proximal tubules of sham operated animals (**B**) Bax protein in the sham-operated animals exclusively on distal tubule (**C**) up-regulated Bcl-2 expression on tubular cells in ARF animals (**D**) widely and strong expression of Bax expended on proximal tubular cells in ARF animals (**E**) slight expression of Bcl-2 protein in group treated with losartan (**F**) reduced Bax protein expression after ischemic-reperfusion injury in losartan treated rats.

Anti-apoptotic Bcl-2 protein expression was absent or very slightly present in proximal tubular cells of SHAM (**Figure 3A**), but it was significantly higher in animals with ARF (**Figure 3C**). Treatment with Losartan markedly reduced the expression of Bcl-2 protein on proximal tubules, indicating lower degree of apoptotic lesions in them (**Figure 3E**).

Bax protein had slightly to moderate expression, mainly on some distal tubules of cortico-medullar zone, in SHAM (**Figure 3B**). The expression of the Bax protein is almost completely absent in the cortex of these animals. However, the kidneys of animals with ARF showed the expression of Bax proteins on some cortical proximal tubules, in addition to intensive expression on distal cortico-medullary tubules (**Figure 3D**). Losartan treatment leads to decrease of Bax protein expression after IRI (**Figure 3F**). There is almost complete absence of Bax protein expression particularly in cortex, with slight presence on distal tubuli.

## Discussion

This study, for the first time, demonstrated that in combined model of hypertension and postischemic ARF, kidney oxidative injuries are strongly mediated by Ang II.

Bayrak et al. point to increased plasma LP in renal IRI [17]. In SHR, the AngII-AT_1_R mediated effects on kidney ROS are associated with NADPH oxidase overexpression, even before the onset of hypertension [18]. Blockade of RAS with angiotensin converting enzyme (ACE) inhibitor, Captopril, [19] decreased oxidative stress of these rats. Also, AT1R antagonist, Candesartan, significantly attenuate LP in humans [20]. Our results are consistent with previously mentioned, because Losartan reduced TBARS, therefore, at least partly, protects target cells from LP injury. Also, the improved antioxidant defense, due to increased CAT activity after losartan treatment, in our study, further suggests that AT1R blockade could attenuate oxidative stress in SHR with induced postischemic ARF. Losartan can also prevent the increased concentration of H_2_O_2_ caused by Ang II infusion [21]. In addition, it was shown that eight-week Losartan treatment leads to an increase of CAT activity in damaged cardiac tissue of Sprague-Dawley rats [22]. It is well-known that hydroxyl radical, one of the most reactive oxygen species, as a product of H_2_O_2_ degradation (Fenton's reaction), has an important role in LP, but CAT decomposes H_2_O_2_ to both electro-neutral H_2_O and O_2_ molecules. In our model of ischemic ARF in SHR, increased CAT activity and reduced LP, due to AT1R antagonism, indicate a close connection between RAS and oxidative stress. On the other hand, activity of other antioxidant enzymes SOD, GR, and GSH-Px were unaffected by ARF or losartan treatment, indicating that the activity of these enzymes is not directly mediated by RAS in the presented experimental setting.

In our study blood pressure was moderately reduced after ARF induction, similar to the results performed in the model of glycerol induced ARF. In this model Bowmer [23] considered high uremia influence on autonomic nervous system (diminished α1 adrenoreceptors sensitivity) as a cause of blood pressure reduction. Further, in the present study losartan as a potent vasodilator additionally decreased MAP in treated rats compared to control ARF. This suggests that Ang II strongly participates in blood pressure control after renal ischemia in SHR. Our results were similar to Miloradović et al. [8] who showed that losartan reduced MAP in L-NAME (3 mg/kg b.m.) induced hypertensive Wistar rats after ARF induction.

Previous results of our group showed that neither ACE inhibitor nor competitive inhibitor of xanthine oxidase were able to ameliorate the decline of glomerular filtration in ARF hypertensive rats [24]. Kontogiannis and Burns [4] showed that blockade of AT1R with Losartan accelerated the recovery of renal function, leading to a significant decrease in serum creatinine of male Sprague-Dawley rats, with 60 minutes bilateral occlusion of renal hilum. Furthermore, Miloradović et al. [8] showed that Losartan led to a slight GFR improvement in postischemic kidney of Wistar rats after moderate blockade of NO synthesis. In the present study, Losartan significantly increased GFR 24 h after renal reperfusion, in addition to previously described haemodynamic benefits [25]. This confirms that Ang II plays a critical role in the regulation of glomerular filtration in disturbed hypertensive vasoactive milieu after IRI. Low C_U_, another marker of altered GFR in ARF, was also ameliorated with losartan.

Protective effects of HDL have been found in animal studies, as well as in patients [10], [26]. Thiemermann et al [10], showed that bolus of HDL could improve renal function and structure after ischemic ARF in Wistar rats. In our study, blockade of AT1R with losartan resulted in a significant increase of plasma HDL. It was shown that Ang II downregulates protective HDL receptors on tubular cells via AT1R [9], therefore, AT1R blockade could have beneficial effects. Furthermore, due to antioxidative properties of AT1R blockade, HDL appears as a main fraction in total plasma cholesterol content.

Morphological changes in the kidney tissue represent the best portrait of ARF development [27], [28]. The most notable changes in the ischemic kidney that can be seen by light microscopy are broad areas of necrosis, the most common in the cortico-medullary zone, brush border loss and a large number of PAS positive casts in the renal medulla [27], [29]. Morphological changes in the kidneys isolated from rats with induced ischemic ARF, exactly correspond to the previous description. In addition to these changes, the dilations of certain segments of the proximal and distal tubules, with or without loss of brush-border were observed.

Our results clearly indicate that Losartan, used to block AT1R in the early stages of ischemic ARF in hypertension, has beneficial effects on renal morphological structure. Lesions of tubular epithelial cells (the main damage occurred during ARF) are less severe in Losartan treated rats. This histological finding suggests a positive effect of Losartan, which is further consistent with the improvement of both systemic and renal artery hemodynamic parameters [25], as well as the biochemical parameters of kidney function improvement in the present study. Heeb [30] showed a protective effect of Losartan on kidney morphology in rats with gentamicin-induced ARF. The other study also showed a beneficial effect of Losartan on the kidney morphology in rats with malignant hypertension [31].

The survival and regeneration of cells are important factors in the recovery of renal function in ARF. The relationship between Bax and Bcl-2 expression can be a good predictor of renal recovery potential after ischemic damage in ARF. The study of Gobe et al. [3] showed the increased expression of Bax and Bcl-2 in the kidney, 24 h after 30 minutes bilateral renal ischemia in Sprague Dawley rats. Bax was increased in both the proximal and distal tubules, and Bcl-2 protein was upregulated in tubular epithelial cell. These results are consistent with the results we obtained in this study, where ischemic ARF leaded to increased expression of Bax in SHR. On the other hand, in Ang II induced-hypertension, Losartan reduced the expression of Bax in the kidney, but did not affect Bcl-2 level [32]. In our experimental study AT1R blockade significantly reduces the expression of Bax, as well as Bcl-2, after IRI. Considering that Ang II-induced apoptosis of mesangial and proximal tubular cells is associated with increased generation of ROS [18], our findings suggest that the RAS blockade slows cell death in postischemic hypertensive kidney, thus helps kidney to establish better function.

## Conclusions

From our results we can conclude that Ang II plays a significant role in the development and maintenance of experimental postischemic ARF in the hypertensive rats and partly contribute to the blood pressure increasing Also, these results suggest that the intensity of tubular injury could be reduced in the hypertensive patients who are on therapy with AT1R blockers, and that these patients have no particular risks of kidney function deterioration during the ischemic ARF episode. However, our hypothesis requires further complex and comprehensive clinical research for definite conclusions about AT1R antagonist usage in ARF hypertensive patients.
